# Physician-staffed helicopter emergency medical service has a beneficial impact on the incidence of prehospital hypoxia and secured airways on patients with severe traumatic brain injury

**DOI:** 10.1186/s13049-017-0438-1

**Published:** 2017-09-15

**Authors:** Toni Pakkanen, Antti Kämäräinen, Heini Huhtala, Tom Silfvast, Jouni Nurmi, Ilkka Virkkunen, Arvi Yli-Hankala

**Affiliations:** 1 FinnHEMS Ltd, Research and Development Unit, Vantaa, Finland; 2 Department of Anaesthesia, Tampere University Hospital, Tampere, Finland; 3 Tays Emergency Medical Service, FinnHEMS 30, Tampere University Hospital, Tampere, Finland; 4 Faculty of Social Sciences, University of Tampere, Tampere, Finland; 5 Department of Anaesthesia and Intensive Care, Helsinki University Hospital, University of Helsinki, Helsinki, Finland; 6 Department of Emergency Medicine and Services, Helsinki University Hospital and Emergency Medicine, University of Helsinki, Helsinki, Finland; 7 Faculty of Medicine and Life Sciences, University of Tampere, Tampere, Finland

**Keywords:** Prehospital emergency care (MeSH), Emergency medical services (MeSH), Critical care (MeSH), Traumatic brain injury (MeSH), Airway management (MeSH), Endotracheal intubation (MeSH), Patient outcome assessment (MeSH), Glasgow outcome scale (MeSH)

## Abstract

**Background:**

After traumatic brain injury (TBI), hypotension, hypoxia and hypercapnia have been shown to result in secondary brain injury that can lead to increased mortality and disability. Effective prehospital assessment and treatment by emergency medical service (EMS) is considered essential for favourable outcome. The aim of this study was to evaluate the effect of a physician-staffed helicopter emergency medical service (HEMS) in the treatment of TBI patients.

**Methods:**

This was a retrospective cohort study. Prehospital data from two periods were collected: before (EMS group) and after (HEMS group) the implementation of a physician-staffed HEMS. Unconscious prehospital patients due to severe TBI were included in the study. Unconsciousness was defined as a Glasgow coma scale (GCS) score ≤ 8 and was documented either on-scene, during transportation or by an on-call neurosurgeon on hospital admission. Modified Glasgow Outcome Score (GOS) was used for assessment of six-month neurological outcome and good neurological outcome was defined as GOS 4–5.

**Results:**

Data from 181 patients in the EMS group and 85 patients in the HEMS group were available for neurological outcome analyses. The baseline characteristics and the first recorded vital signs of the two cohorts were similar. Good neurological outcome was more frequent in the HEMS group; 42% of the HEMS managed patients and 28% (*p* = 0.022) of the EMS managed patients had a good neurological recovery. The airway was more frequently secured in the HEMS group (*p* < 0.001). On arrival at the emergency department, the patients in the HEMS group were less often hypoxic (*p* = 0.024). In univariate analysis HEMS period, lower age and secured airway were associated with good neurological outcome.

**Conclusion:**

The introduction of a physician-staffed HEMS unit resulted in decreased incidence of prehospital hypoxia and increased the number of secured airways. This may have contributed to the observed improved neurological outcome during the HEMS period.

**Trial registration:**

ClinicalTrials.gov IDNCT02659046. Registered January 15th, 2016.

## Introduction

After traumatic brain injury (TBI), hypotension, hypoxia and hypercapnia have been shown to result in secondary brain injury that can lead to increased mortality and disability [1]. As the prognosis of patients with severe TBI and a low Glasgow Coma Scale (GCS) score depends on early support of vital functions [2, 3], effective prehospital assessment and treatment is considered essential for favourable outcome [4]. In particular, prehospital prevention of hypoxia by adequate airway and respiratory management including a secured airway, normoventilation and prevention of aspiration is strongly associated with improved outcome [5–8].

Depending on the structure of the emergency medical service (EMS) system the level of available treatment varies, and this may have an impact on the patient’s outcome. A systematic review from 2009 revealed only a few controlled studies examining the effect of advanced interventions by a prehospital EMS physician on outcome. Increased survival was found in major trauma patients and in patients with cardiac arrest [9].

Although a Helicopter Emergency Medical Service (HEMS) is a part of the prehospital trauma system in many countries, HEMS and the possible impact it has on outcome in traumatically injured patients remains a subject of debate. Studies have been performed with the aim to evaluate the effect of HEMS on outcome in trauma patients, with contradictory results [10–15]. Differences in HEMS team composition, dispatch protocols, EMS organisation, hospital treatment and methodology and outcome measures make comparisons between studies difficult.

The aim of this study was to evaluate the effect of a physician-staffed HEMS in the treatment of TBI patients. The hypothesis was that implementation of a physician-staffed HEMS would have a positive effect on outcome.

## Material and methods

The Pirkanmaa district has the second largest population in Finland, with approximately a half million inhabitants living in the city of Tampere and in the surrounding municipalities. All TBI patients in the study region are admitted to Tampere University Hospital, which is the referral centre in the area, and provides immediate neurosurgical care according to national guidelines (the first edition published in 2003, with an update in 2008) [16].

### Period 1 (2005–2010): Paramedic EMS (EMS group)

The EMS was the responsibility of and organised by each of the municipalities in the region. The system was two-tiered, with emergency medical technician basic life support and paramedic advanced life support units. There were no dedicated on-call EMS medical directors, and no physician-staffed EMS units available on-scene. Prehospital crews consulted on-call hospital and local primary care physicians for treatment guidelines when deemed necessary. Patients with a decreased level of consciousness were routinely administered oxygen according to national guidelines and ventilation was assisted with bag-valve mask if required. Endotracheal intubation was primarily performed in cardiac arrest patients and infrequently in patients with a decreased level of consciousness. Hypnotics or neuromuscular blocking agents were not available in the prehospital setting and endotracheal intubation was performed using sedatives and opioids only, at the discretion of the paramedic on the scene.

### Period 2 (2012–2015): Physician-staffed HEMS (HEMS group)

A physician-staffed HEMS was introduced into the EMS in the autumn of 2011, covering all municipalities in the study area. The HEMS is dispatched on primary missions together with basic or advanced life support EMS units to patients with potential major trauma or other critical medical condition. The role of the helicopter is primarily to transport the physician to the scene, while patient transport is mainly carried out by EMS ground vehicles with the physician escorting the patient to the emergency department (ED) when necessary. The physicians are anaesthesiologists experienced in prehospital critical emergency medicine and conduct advanced airway management according to the HEMS unit TBI standard operation procedure (SOP). General anaesthesia complying with the principles of neuroanaesthesia, including hypnotics, opioids and neuromuscular blocking agents, is routinely used for rapid sequence intubation (RSI). Capnography-assisted controlled ventilation, invasive haemodynamic monitoring with arterial blood gas sampling and, if necessary, noradrenaline-infusion and hypertonic saline are also routinely employed according to the unit’s TBI SOP and national guidelines [16].

### Study design

This retrospective cohort study compares the outcome of patients with severe TBI. Prehospital data from two periods were collected: before (EMS group) and after (HEMS group) the implementation of the HEMS. Data of the EMS group have been presented in a previous study [10] comparing the outcome of TBI patients in two differently structured EMS systems and were used as a historical control cohort in the current study. As the physician-staffed HEMS was introduced in the autumn of 2011, that year was excluded from data collecting.

Unconscious prehospital patients due to severe TBI were included in the study. Unconsciousness was defined as a Glasgow coma scale (GCS) score ≤ 8 [17] and was documented either on-scene, during transportation or by an on-call neurosurgeon on hospital admission. The ICD-10 hospital discharge diagnoses for traumatic brain injury and/or skull fracture (S06.2-S06.6, S06.8, S02.1) were used to identify the patients, and their patient records were cross-referenced with EMS and HEMS run-sheets. Patients with concomitant multiple injuries with the need for other than neurosurgical interventions were excluded, as were patients transferred from other hospitals.

Data collected included age, gender, mechanism of injury, GCS score and vital signs on-scene and on arrival at the ED, airway management, response and total mission times. Hypoxia was defined as an SpO_2_ below 90% and hypotension was defined as a systolic blood pressure (SBP) below 90 mmHg. These definitions are consistent with the latest edition of the Brain Trauma Foundation’s guidelines for prehospital management of traumatic brain injury [1]. For assessment of neurological outcome, a modified six-month Glasgow Outcome Score (GOS) was used [18, 19]. A GOS of 1 denoted death within six months, GOS 2–3 poor neurological outcome (need for assistance in activities of daily living) and GOS 4–5 good neurological recovery (independent life). Outcome evaluation was performed, or time of death was obtained, by the corresponding author, based on hospital patient records six months after the incident. If the outcome evaluation was unclear, the research team members reviewed the case and a joint decision was made.

### Statistical analyses

Results are expressed as medians with ranges or percentages. The groups were compared using the chi-square or Fisher’s exact test for categorical variables. Six-month survival is presented with Kaplan-Meier curves. Comparison between EMS and HEMS was made with the log-rank test.

Binary logistic regression analysis was used to predict good outcome. Variables of the univariate analysis with *p* < 0.05 were added to the multivariable analysis. Statistical significance was considered at a *p*-value less than 0.05. The data were analysed using IBM SPSS Statistics for Windows Version 21.0. Armonk, NY: IBM Corp. released 2012.

## Results

**Fig. 1:**
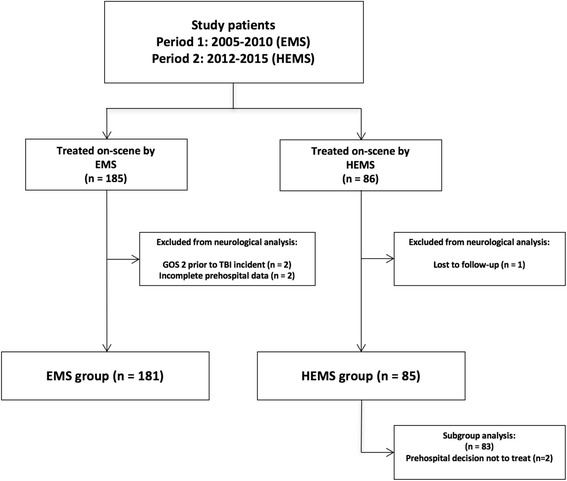
Flow-chart

During the study period (Periods 1 + 2) data from 181 patients in the EMS group and 85 patients in the HEMS group were available for neurological outcome analyses (Fig. 1). The baseline characteristics and the first recorded vital signs of the two cohorts were similar and are presented in Table 1.

**Table 1:** Baseline characteristics

		Period 1: EMS		Period 2: HEMS		
		*n* = 181		*n* = 85		p-value
Age, years	(Median, Q_1_-Q_3_)	54	33–69	53	23–74	0.479
Gender, male	(n, %)	127	70	58	68	0.820
Mechanism of injury	(n, %)					0.288
Fall from ground level		79	43	32	38	
Traffic accident		41	23	29	34	
Fall from a height (> 2 m)		25	14	9	11	
Violence		14	8	6	7	
Other		4	2	4	5	
Unknown		18	10	5	6	
Primary GCS	(Median, Q_1_-Q_3_)	5	3–7	5	3–7	0.956
Primary vital parameters	(n/total, %)					
Hypoxia		32/170	19	18/84	21	0.369
Hypotension		7/174	4	4/84	5	0.514
Airway secured	(n, %)	29	16	81	95	< 0.001
Prehospital decision not to treat		–	–	2	2	
Vital parameters on arrival at the ED	(n/total, %)					
Hypoxia		18/173	10	2/84	2	0.024
Hypotension		7/179	4	4/85	5	0.750
Mission related times, minutes	(Median, Range)					
From dispatch to arrival on-scene						
1st EMS Unit on-scene		8	0–37	12	4–41	0.006
HEMS		–	–	23	6–85	
Total mission time		54	18–180	82	30–201	< 0.001

**Fig. 2:**
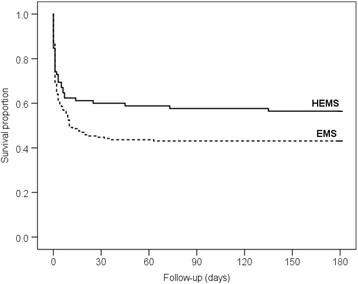
Six-month survival according to EMS system (Log Rank *p* = 0.066)

Good neurological outcome was more frequent in the HEMS group; 42% of the HEMS managed patients and 28% (*p* = 0.022) of the EMS managed patients had a good neurological recovery (GOS 4–5), living an independent life six months after the incident. There was a trend to higher survival (53% vs. 43%, Log Rank *p* = 0.066) in the HEMS group during the 6-month follow-up period, presented as Kaplan-Meier curves in Fig. 2. A prehospital decision not to treat was done by the attending HEMS physician on two patients. A subgroup Kaplan-Meier analysis with these two patients removed from the HEMS group resulted in higher survival (*p* = 0.045).

The logistic regression analysis is presented in Table 2. The airway was secured more frequently in the HEMS group (*p* < 0.001). Due to long distances, 10 patients were air transported to the ED in the HEMS group, while patient transport was mainly carried out by EMS ground vehicles with the physician escorting the patient. On arrival at the ED, patients in the HEMS group were less often hypoxic (*p* = 0.024). In univariate analysis HEMS-period, lower age and secured airway were associated with good neurological outcome. In multivariable analysis lower age remained as a significant factor for good outcome.

**Table 2:** Univariate and multivariable logistic regression of six-month good outcome predictors

	Univariate			Multivariable		
	OR	95% CI	p-value	OR	95% CI	p-value
Period						
HEMS	1.87	1.09–3.21	0.022	2.46	0.89–6.84	0.083
EMS	1					
Age	0.95	0.93–0.96	< 0.001	0.95	0.93–0.96	< 0.001
Sex						
Male	1.78	0.99–3.19	0.055	Not entered		
Female	1					
GCS	1.07	0.97–1.18	0.183	Not entered		
Hypoxia						
On-scene	1			Not entered		
Not present	1.65	0.81–3.37	0.165			
Hypotension						
On-scene	1			Not entered		
Not present	2.26	0.48–10.72	0.303			
Airway						
Secured	1.89	1.12–3.19	0.017	0.71	0.27–1.88	0.486
Not secured	1					
Hypoxia						
At ER	1			Not entered		
Not present	2.12	0.69–6.54	0.193			
Hypotension						
At ER	1			Not entered		
Not present	2.28	0.48–10.78	0.299			

## Discussion

The introduction of a physician-staffed HEMS unit significantly decreased the proportion of hypoxic TBI patients and increased the number of patients with secured airways on hospital admission. This may have contributed to the observed improved neurological outcome during the HEMS period.

This supports our previous findings when evaluating mortality and neurological outcome of TBI patients in two regions with differently structured EMS systems [10].

The baseline characteristics of the two cohorts were similar, with no differences between the groups regarding gender, mechanism of injury or initial GCS. Only the mission-related time frames differed, since the response time and delay to hospital admission were longer in the HEMS group. In the HEMS group 10 patients were air transported to the ED, but as the total mission times in the HEMS group were longer, this result in our opinion excludes the impact of the air transport itself.

The principles for prehospital airway management of TBI patients are described in international guidelines: an airway should be established in patients who have severe TBI (GCS ≤ 8), who are unable to maintain an adequate airway or who are hypoxaemic despite supplemental oxygen [1]. The optimal way to secure the airway still remains controversial [20, 21]. With endotracheal intubation, if RSI is performed poorly, hypoxia and hypotension have been shown to have a negative effect on outcome of TBI patients [21–23]. In the present study, virtually all patients were intubated in the prehospital setting in the physician-staffed HEMS group, whereas only a few patients were intubated in the paramedic EMS group. In univariate analysis of the HEMS-period, securing the airway was associated with good neurological outcome. Anaesthetics were used by the HEMS physicians, while the paramedics were limited to the use of sedatives and opioids. This may have influenced on the observed difference in the rate of airway management procedures during the two periods.

In previous studies both hypoxaemia and hypotension have been shown to have a negative impact on TBI outcome [2, 5]. We found no difference in the on-scene occurrence of disturbances of these vital signs between the study groups. The proportion of hypotensive patients on arrival to the ED was similar in both groups. However, hypoxia was more common in the patients managed by the paramedic EMS. The likely explanation for this finding is the higher frequency of prehospital endotracheal intubation, controlled ventilation and more precise and invasive monitoring of the vital signs in the HEMS group.

Age has been demonstrated to be an important predictor of outcome after head injury. Older age has been shown to be an independent risk factor for higher mortality and poor functional outcome in TBI [24, 25]. In this study, in uni- and multivariate analysis of the HEMS-period lower age was associated with good neurological outcome.

## Study limitations

This was a retrospective observational study and the following limitations should be considered when interpreting the results. The prehospital data were originally self-reported, could not be independently verified, and could therefore have been biased. Continuous data on vital signs covering the whole prehospital phase were not available; therefore, short-lived hypoxia or hypotension during the prehospital period cannot with certainty be excluded during either period. Due to the low rate of endotracheal intubation in the paramedic EMS group, parameters regarding ventilation could not be compared. The first CT scans were not evaluated using the Marshall classification. Neurosurgical and intensive care have advanced during the study period, which may also have affected the results and may to some extent account for the improved outcome. Outcome evaluation was based on patient record assessment without clinical examination or the help of a questionnaire. It is possible that the deaths occurring in the late stages of the follow-up period were unrelated to the prehospital index event, with secondary diseases or injury being the cause.

## Conclusions

The introduction of a physician-staffed HEMS unit resulted in a beneficial impact on patient care reflected by a decreased incidence of prehospital hypoxia and an increased number of patients with secured airways. This may have contributed to the observed improved neurological outcome during the HEMS period. Further prospective multicentre studies with detailed data are needed to confirm the hypothesis that a physician-staffed HEMS has a positive impact on the outcome of TBI patients.

## Abbreviations

ED: Emergency Department
EMS: Emergency Medical Services
GCS: Glasgow Coma Scale
GOS: Glasgow Outcome Score
HEMS: Helicopter Emergency Medical Service
RSI: Rapid Sequence Intubation
TBI: Traumatic Brain Injury
